# Case Report: Metachronous bilateral primary and secondary angiosarcoma of the breast

**DOI:** 10.3389/fonc.2026.1883637

**Published:** 2026-09-17

**Authors:** Shulian Zhuang, Jianxing Zhang, Miao Chen, Yunsi Lai, Ling Chen

**Affiliations:** Department of Ultrasound, The Second Affiliated Hospital of Guangzhou University of Chinese Medicine, Guangzhou, China

**Keywords:** angiosarcoma, breast neoplasms, case reports as topic, multiple primary, neoplasms, second primary

## Abstract

**Background:**

Breast angiosarcoma (AS) is an extremely rare and aggressive malignancy with no standardized treatment guidelines. We report an exceptional case of metachronous bilateral primary and secondary AS in a single patient, highlighting the diagnostic and therapeutic challenges posed by this disease.

**Case presentation:**

A patient underwent surgical dissection for primary AS of the right breast. Seven months later, a primary AS was identified in the left breast. Four months after that, a secondary angiosarcoma also developed within the previously irradiated left chest wall and axilla. Postoperative pathology confirmed angiosarcoma in all resected specimens. Following multidisciplinary team discussion, the patient received simple mastectomy, adjuvant chemotherapy, and radiotherapy. Despite this multimodal approach, contrast-enhanced CT follow-up revealed rapid disease progression with distant metastases to the lungs, liver, spleen, bilateral adnexa, and bones. The patient ultimately died of respiratory failure secondary to severe pulmonary infection and atelectasis 22 months after the initial diagnosis.

**Conclusion:**

This extraordinarily rare case of sequential bilateral primary and secondary breast AS underscores the aggressive biological behavior of this malignancy and the limitations of current therapeutic strategies. It highlights the urgent need for identifying specific diagnostic biomarkers and novel therapeutic targets to enable early detection and improve the dismal prognosis associated with this disease.

## 1 Introduction

Breast angiosarcoma (AS) is an extremely rare mesenchymal malignancy, accounting for less than 1% of all breast tumors and associated with a poor prognosis (1). Based on etiology, breast AS is classified into two subtypes: primary angiosarcoma, which arises *de novo* with no identifiable risk factors, and secondary angiosarcoma, which typically occurs following previous breast irradiation. Due to its nonspecific clinical presentation and imaging features, primary breast AS is frequently misdiagnosed, often leading to delayed treatment. Herein, we describe a rare and challenging case of a patient diagnosed with metachronous bilateral primary and secondary angiosarcoma of the breast, with the aim of enhancing the understanding and clinical management of this aggressive disease.

## 2 Case presentation

### 2.1 Timeline of key events

(The chronological sequence of major events is summarized in **Figure 1** and detailed below):

**Figure 1:**
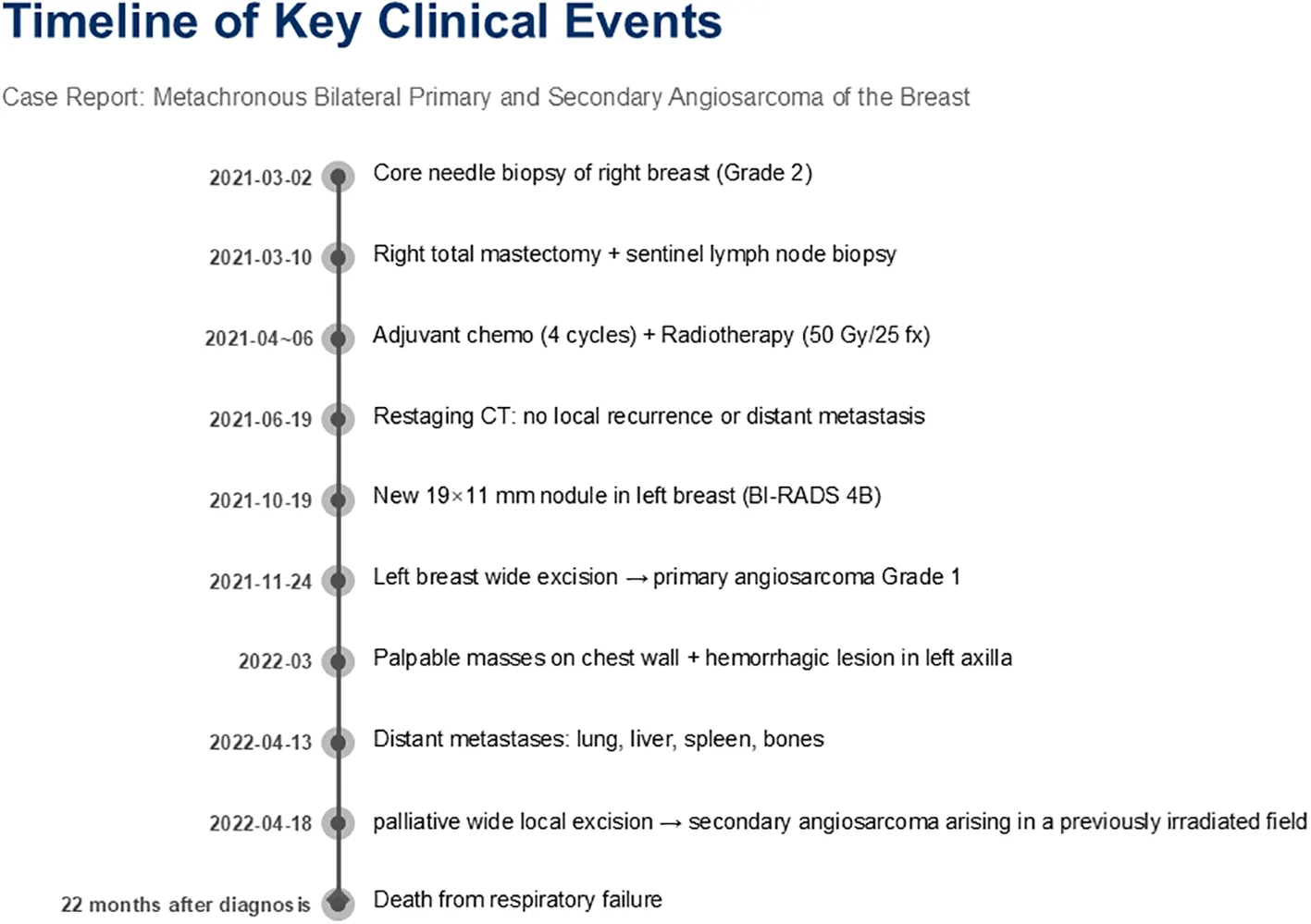
Flowchart summarizing the patient’s clinical timeline. This figure illustrates the chronological sequence of key diagnostic, therapeutic, and clinical events from the initial presentation (March 2021) to the patient’s death (February 2023). Major milestones include: right breast biopsy and mastectomy (March 2021), adjuvant chemotherapy and radiotherapy (April–June 2021), detection of left breast primary angiosarcoma (October–November 2021), diagnosis of secondary angiosarcoma in the irradiated field (March–April 2022), widespread distant metastases (April 2022), and death from respiratory failure (22 months after initial diagnosis). (Figure created by the authors).

- March 2, 2021 – Core needle biopsy of right breast mass.
- March 10, 2021 – Right total mastectomy + sentinel lymph node biopsy.
- April–June 2021 – Adjuvant chemotherapy (ifosfamide+epirubicin, 4 cycles) and radiotherapy (50 Gy/25 fractions) to right chest wall.
- June 19, 2021 – Restaging CT: no local recurrence or distant metastasis.
- October 19, 2021 – New nodule detected in left breast (19×11 mm).
- November 24, 2021 – Local extended excision of left breast mass; pathology confirmed primary angiosarcoma (Grade 1).
- March 2022 – Palpable masses on anterior chest wall and hemorrhagic lesion in left axilla.
- April 13, 2022 – Subsequent staging CT – Distant metastases to lungs, liver, spleen, bilateral adnexa, right third anterior rib, and T11 vertebral body.
- April 18, 2022 – palliative wide local excision of chest wall and left axillary skin lesions; pathology confirmed secondary angiosarcoma arising in a previously irradiated field.
- 22 months after initial diagnosis – Patient died of respiratory failure secondary to severe pulmonary infection and atelectasis.

### 2.2 Initial presentation and diagnosis (right breast)

A 41-year-old female presented with a 6-month history of a progressively enlarging, painless, palpable mass in her right breast. Physical examination revealed asymmetric breast enlargement with a diffuse, ill-defined mass occupying the upper outer quadrant, measuring approximately 90 mm × 90 mm. The overlying skin exhibited erythematous changes spanning roughly 100 mm × 100 mm, with normal local temperature. No nipple discharge, peau d’orange, or skin dimpling was observed. There was no palpable axillary lymphadenopathy.

Breast ultrasound demonstrated a diffuse, inhomogeneous, hypoechoic lesion with indistinct borders and internal vascularity, classified as BI-RADS category 4B (**Figures 2a, b**). Mammography revealed a mass with associated skin thickening in the upper outer quadrant, categorized as BI-RADS 4C (**Figures 2c, d**). Contrast-enhanced chest CT identified a soft tissue mass suspicious for malignancy (**Figures 2e, f**) and right axillary lymph node enlargement. Whole-body bone SPECT/CT showed no evidence of osseous metastasis.

**Figure 2:**
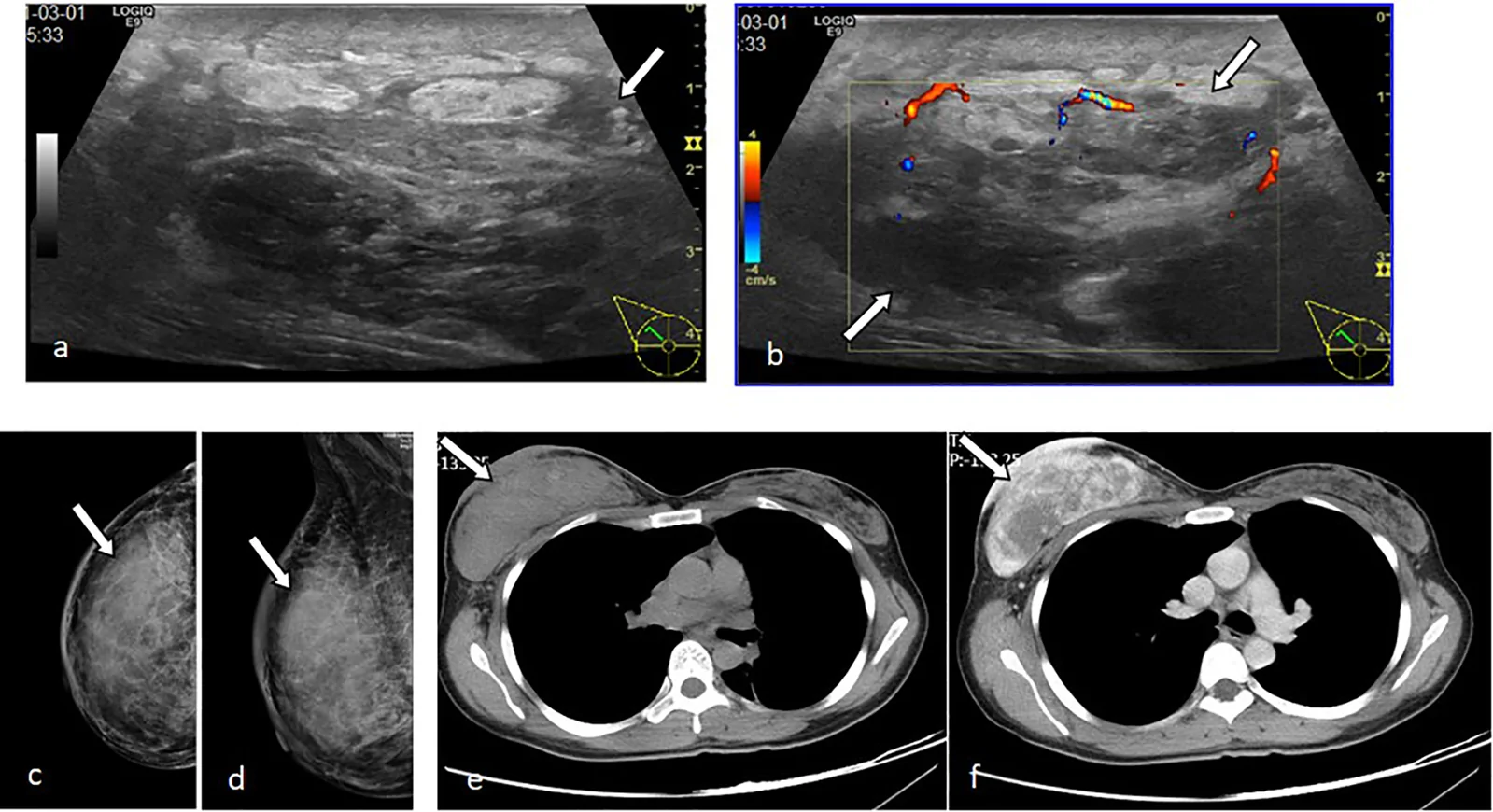
**(a–f)** Preoperative imaging of the right breast primary angiosarcoma. **(a, b)** B-mode ultrasound and color Doppler showing a diffuse, inhomogeneous, hypoechoic lesion with poorly defined borders and internal vascularity (arrows). **(c, d)** Mammography [CC view **(c)** MLO view **(d)**] revealing focal asymmetric density and skin thickening (arrows). **(e, f)** Contrast-enhanced CT demonstrating an ill-defined soft-tissue mass with inhomogeneous enhancement and overlying skin thickening (arrows).

On March 2, 2021, a core needle biopsy of the right breast mass, along with a skin punch biopsy, was performed. Pathological examination revealed a well-differentiated angiosarcoma (Grade 2), with dermal infiltration noted in the skin specimen.

At the time of initial presentation, clinical examination and imaging (mammography and ultrasound) of the contralateral left breast revealed no suspicious findings.

### 2.3 First treatment and clinical course

On March 10, 2021, the patient underwent a right total mastectomy with skin flap reconstruction and sentinel lymph node biopsy. Histopathological analysis confirmed a 100 mm × 85 mm × 35 mm angiosarcoma in the upper outer quadrant. Dermal involvement was present without epidermal invasion, and surgical margins were clear (≥2.0 mm). Sentinel lymph nodes (0/2) were negative for malignancy. Immunohistochemical staining revealed the following profile: ER (5% weakly positive), PR (5% weakly positive), HER2 negative, Ki67 (hot spot ~30%), GATA-3 negative, SMMHC positive, P63 negative, pan-cytokeratin negative, CD31 positive, ERG positive, and SMA positive (**Figures 3a–c**).

**Figure 3:**
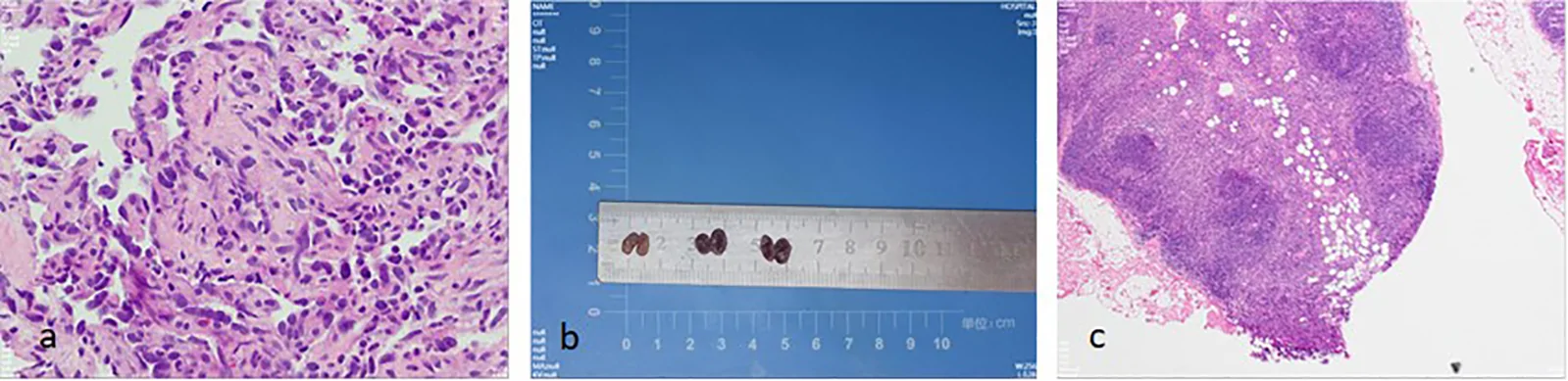
**(a–c)** Histopathology of the primary right breast angiosarcoma. **(a)** Microscopic examination (hematoxylin and eosin [H&E] stain, ×100) showing a well-differentiated angiosarcoma with dermal infiltration. **(b)** Gross specimens of the right axillary lymph nodes. **(c)** H&E stain (×10) of the sentinel lymph node confirming no tumor involvement (0/2).

Following multidisciplinary consultation, the patient received adjuvant chemotherapy with four cycles of ifosfamide and epirubicin (April 2 to June 14, 2021), complicated by grade IV bone marrow suppression with fever. Concurrent radiotherapy to the right chest wall (50 Gy in 25 fractions) was administered from April 20 to June 14, 2021. A restaging chest CT on June 19, 2021, showed no evidence of local recurrence or distant metastasis.

### 2.4 Left breast primary angiosarcoma

On October 19, 2021, a routine surveillance contrast-enhanced CT revealed a new 19 mm × 11 mm nodule in the lower inner quadrant of the left breast. Subsequent mammography (BI-RADS 4B), ultrasound (BI-RADS 4B), and MRI (BI-RADS 4A) confirmed a suspicious lesion (**Figures 4a–g**). On November 24, 2021, the patient underwent a local extended excision of the left breast mass. Pathology confirmed a primary, well-differentiated angiosarcoma (Grade 1), with immunohistochemistry showing CD31+, ERG+, SMA+, CK-, and Ki67 (20%) (**Figures 5a–c**). Postoperatively, adjuvant chemotherapy and radiotherapy were not administered. This decision was made after multidisciplinary discussion, considering that the patient had recently completed extensive multimodal treatment (chemotherapy and radiotherapy) for the contralateral right breast primary, and there is currently no established evidence supporting the routine use of adjuvant systemic or radiation therapy for primary breast angiosarcoma. The patient was subsequently maintained on active surveillance with regular clinical and imaging follow-up. “

**Figure 4:**
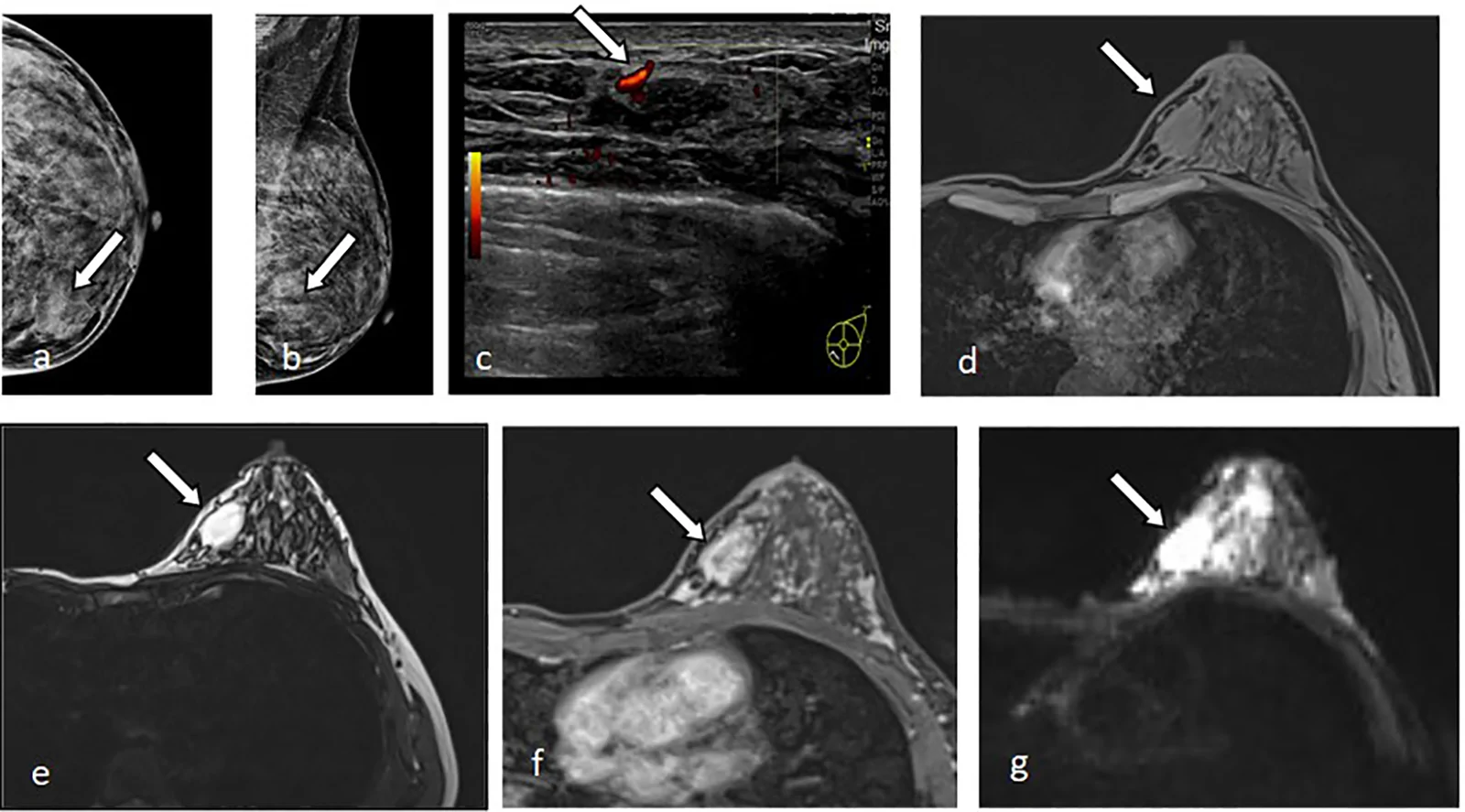
**(a–g)** Imaging of the left breast primary angiosarcoma. **(a, b)** Mammography (CC view a; MLO view b) showing an irregular nodule with indistinct margin (arrows). **(c)** Ultrasound demonstrating a partially angular, microlobulated mass with peri-lesional blood flow (arrow). **(d–g)** MRI: **(d)** fat-suppressed T1-weighted image – low signal; **(e)** T2-weighted image – high signal; **(f)** dynamic contrast-enhanced MRI – heterogeneous enhancement (arrow); **(g)** diffusion-weighted image – diffusion restriction (arrow).

**Figure 5:**
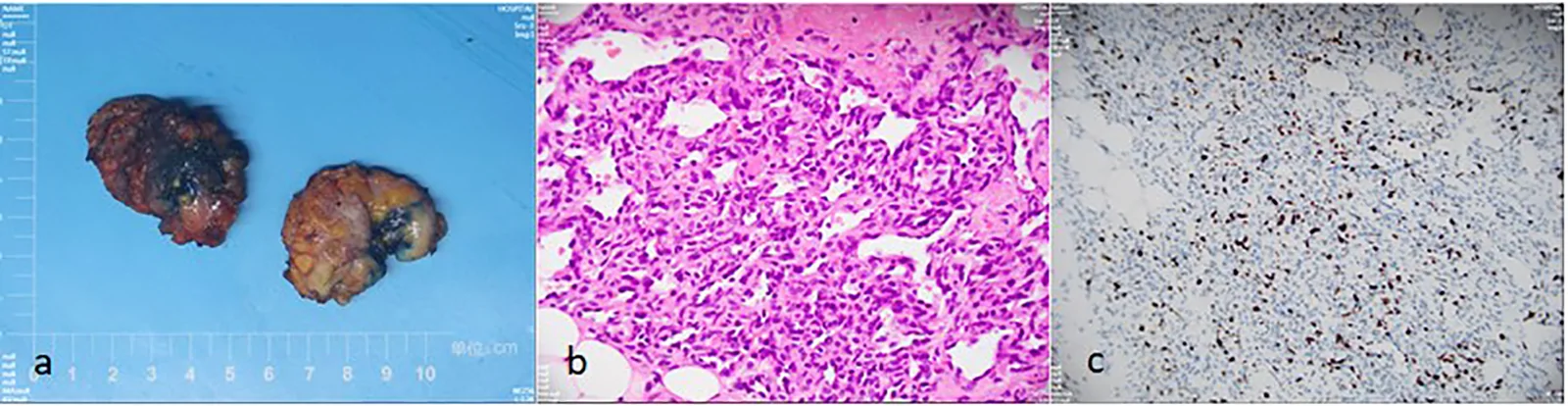
**(a–c)** Gross and microscopic pathology of the left breast primary angiosarcoma. **(a)** Gross specimen showing an ill-defined, soft, hemorrhagic tumour. **(b)** H&E stain (×40) confirming a well-differentiated primary angiosarcoma (Grade 1). **(c)** Immunohistochemistry (×100): CD31+, ERG+, SMA+, CK–, Ki-67 20%.

### 2.5 Chest wall and axillary secondary angiosarcoma

In March 2022, the patient presented with new palpable masses on the anterior chest wall and a hemorrhagic lesion in the left axilla (**Figures 6a, b**). Ultrasound raised suspicion for secondary angiosarcoma (**Figures 6c, d**). On April 18, 2022, she underwent palliative wide local excision of the chest wall and left axillary skin lesions. The primary objectives of the procedure were to control local symptoms, including pain and recurrent bleeding from the friable tumors, to prevent secondary infection, and to obtain tissue for further pathological confirmation. The decision to proceed with surgery, despite the presence of distant metastases, was made after careful multidisciplinary discussion, weighing the potential benefits of local disease control against the patient’s overall performance status and the limited systemic treatment options available. “ Histopathology confirmed secondary angiosarcoma arising in a previously irradiated field, demonstrating an infiltrative vasoformative neoplasm within the dermis and superficial subcutis (**Figures 6e–g**).

**Figure 6:**
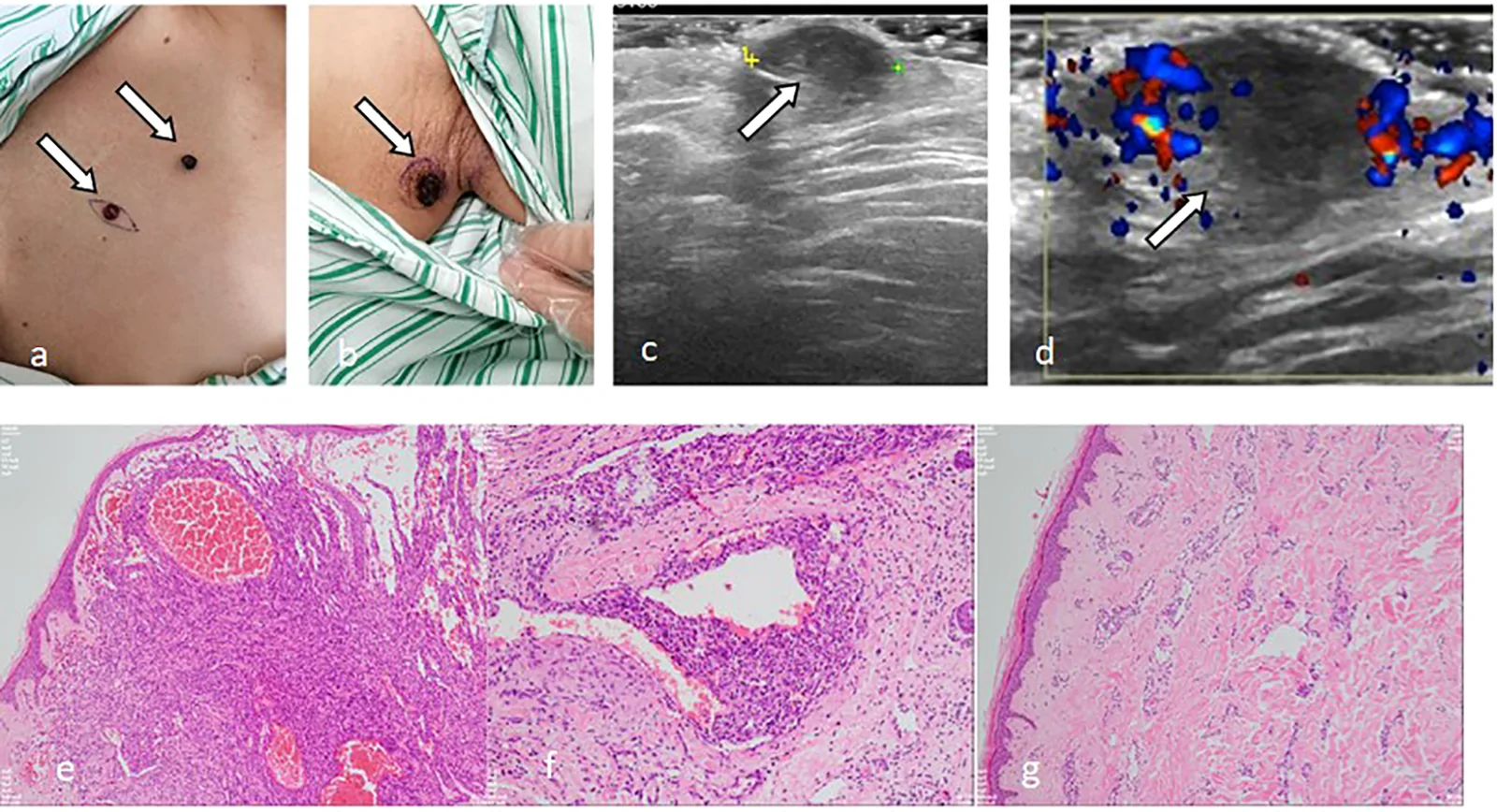
**(a–g)** Clinical and pathological findings of the secondary angiosarcoma. **(a, b)** Physical examination showing erythematous, friable cutaneous nodules on the anterior chest wall and left axilla (arrows). **(c, d)** Ultrasound revealing hypoechoic nodules with abundant colour Doppler flow within the dermis (arrows). **(e–g)** H&E sections (**e, f** from chest wall; **g** from axilla, ×40) demonstrating an infiltrative vasoformative neoplasm in the dermis and superficial subcutis.

### 2.6 Disease progression and outcome

On April 13, 2022, a subsequent contrast-enhanced CT revealed distant metastases to the lungs, liver, spleen, bilateral adnexa, right third anterior rib, and T11 vertebral body (**Figures 7a–d**). Despite ongoing management, the patient’s condition deteriorated. She ultimately died of respiratory failure secondary to severe pulmonary infection and atelectasis, 22 months after her initial diagnosis.

**Figure 7:**
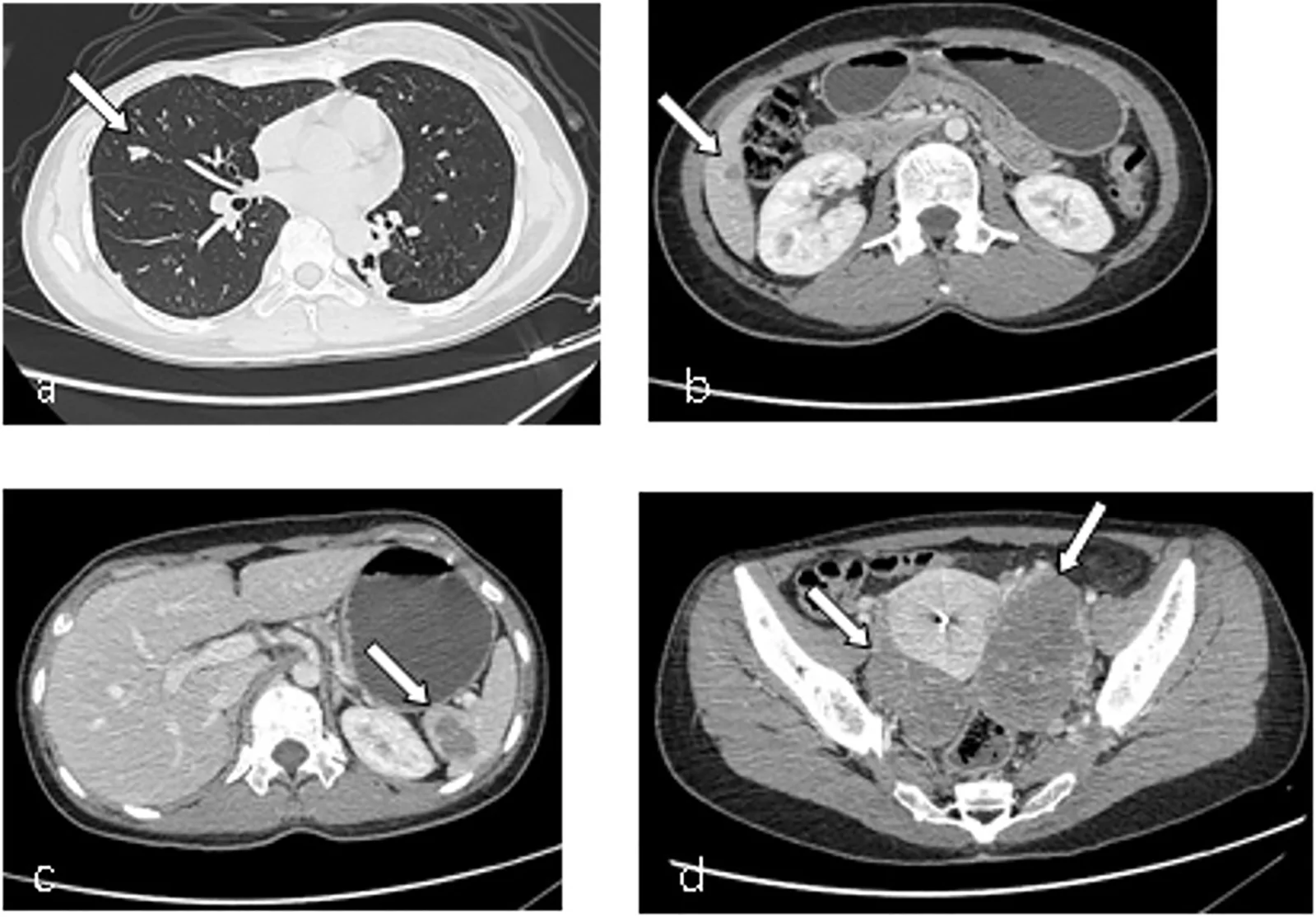
**(a–d)** Post-mortem imaging evidence of widespread haematogenous metastases. Contrast-enhanced CT showing metastatic lesions in **(a)** the lung, **(b)** the liver, **(c)** the spleen, and **(d)** the bilateral adnexal region (arrows).

## 3 Discussion

The clinical significance of this case lies in its exceptional rarity: the development of a primary angiosarcoma in the left breast just seven months after resection of a primary tumor in the right, followed by a secondary angiosarcoma only four months later. This unique timeline of bilateral, metachronous primary and secondary tumors provides critical insights into the unpredictable biological behavior of mammary angiosarcomas. Furthermore, the patient’s rapid deterioration and widespread metastases despite multidisciplinary intervention offer a valuable clinical lesson in the limitations of current therapeutic strategies for advanced-stage disease.

### 3.1 Clinical implications and reflections

The present case offers several important clinical lessons beyond its sheer rarity.

First, the extraordinarily short interval of 9–10 months between the completion of radiotherapy and the detection of the secondary angiosarcoma in the irradiated field challenges the conventional understanding of radiation-associated sarcomagenesis, which typically requires a latency of 6–9 years. While the definitive etiology of this secondary tumor cannot be established, we hypothesize that this unusually short latency may be indicative of an underlying genetic susceptibility or a unique tumor microenvironment that markedly accelerates radiation-induced oncogenesis in this patient. This case, therefore, may represent a distinct, aggressive subtype of secondary angiosarcoma, highlighting the need for further molecular investigation into host factors that influence individual susceptibility to radiation-induced malignancies.

Second, the rapid sequential development of three distinct angiosarcomas — bilateral primary tumors and a subsequent secondary lesion — within a 22-month period suggests an aggressive systemic predisposition in this patient that transcends the local effects of radiation.

Third, the failure of multimodality therapy (surgery, chemotherapy, and radiotherapy) to prevent early and widespread hematogenous dissemination underscores the urgent need for more effective systemic agents. Emerging therapies such as anti-angiogenic tyrosine kinase inhibitors (e.g., pazopanib) and immune checkpoint inhibitors have shown anecdotal activity in sarcomas, and their evaluation in angiosarcoma, particularly in the adjuvant setting, warrants further investigation.

Finally, this case highlights the importance of heightened surveillance in patients with a history of breast angiosarcoma, as contralateral and ipsilateral lesions can develop within a much shorter timeframe than typically anticipated.

### 3.2 Strengths and limitations

This is, to our knowledge, the first reported case of metachronous bilateral primary and secondary angiosarcoma of the breast. The main strength lies in the detailed, longitudinal documentation from initial diagnosis to autopsy-confirmed outcome, supported by comprehensive imaging, histopathology, and immunohistochemistry. A limitation is the single-patient nature of a case report, which precludes generalization of treatment efficacy. Additionally, we were unable to perform molecular analyses (e.g., C-MYC amplification or H3K27me3 loss) that could have further distinguished primary from secondary angiosarcoma.

“We acknowledge that the 7-month interval between the detection of the right and left breast tumors lies near the conventional 6-month threshold used to differentiate synchronous from metachronous breast malignancies. While some might consider this interval compatible with synchronous disease, we have elected to define it as metachronous because it underscores the sequential, independent emergence of two primary tumors in this patient. This is particularly relevant as it facilitates the distinction between the initial primary tumor and the subsequent primary and secondary lesions that developed in a staggered fashion.”

### 3.3 Comparison with the existing literature

#### 3.3.1 Etiology and clinical manifestations

Primary breast angiosarcoma (PBA) arises *de novo* in unirradiated breast parenchyma, rarely involves the skin, and accounts for only 0.04% of breast malignancies and 8% of breast sarcomas (2, 3). Its etiology remains unknown, and it tends to occur in young women, with a mean age at onset of 40 years. The vast majority of PBA cases are unilateral (4, 5). In contrast, secondary breast angiosarcoma (SBA) typically originates in the dermis and subcutaneous tissue of the irradiated breast or in the setting of chronic lymphedema following treatment for another malignancy (6). In the present case, the initial right breast mass and the subsequent left breast mass both arose within the breast parenchyma with associated skin changes, in the absence of prior malignancy or radiotherapy — consistent with a diagnosis of PBA. However, the later skin lesions on the chest wall and left axilla, occurring in a previously irradiated field, are best classified as SBA.

#### 3.3.2 Pathological features

Preoperative diagnosis of angiosarcoma remains challenging (7). Surgical excision with adequate sampling for histopathological and immunohistochemical evaluation is essential (8). The differential diagnosis includes benign hemangioma, invasive breast carcinoma, pseudoangiomatous stromal hyperplasia, and metastatic sarcoma. Immunohistochemical markers such as CD31, CD34, and factor VIII are commonly used (9). Although PBA and SBA share similar gross and histological features, SBA more frequently involves the skin, exhibits higher tumor grade, and is associated with C-myc oncogene amplification (10). Recently, loss of H3K27me3 expression has been proposed as a potential diagnostic marker for radiation-associated angiosarcomas (11). Lymph node metastasis is exceedingly rare; consistent with this, the present case showed no lymph node involvement but demonstrated widespread hematogenous metastases.

#### 3.3.3 Radiologic features

On mammography, angiosarcoma often appears as an indistinct mass, with about 33% being occult in dense breasts (12). Ultrasound typically shows a poorly defined, heterogeneous nodular mass with increased vascularity (13). MRI offers superior soft tissue resolution, typically demonstrating low T1 and high T2 signal intensity, with rapid initial enhancement and washout on dynamic contrast imaging (14, 15). The presence of hyperintense areas on T1-weighted images, corresponding to hemorrhagic foci or vascular lakes, is a characteristic finding that may help differentiate AS from inflammatory breast conditions.

#### 3.3.4 Treatment and prognosis

Surgical excision remains the mainstay, with mastectomy considered standard. Routine axillary lymph node dissection is not indicated (16). Sentinel lymph node biopsy may be considered in selected cases (17). The role of adjuvant radiotherapy and chemotherapy remains controversial; some studies suggest that adjuvant chemotherapy may reduce local recurrence and improve survival (18). A recent meta-analysis identified older age, larger tumor size, higher histological grade, and positive margins as predictors of poor prognosis (19). In the present case, despite multimodal treatment, the patient developed rapid local recurrence and distant metastases, underscoring the limitations of current therapies.

## 4 Take-away lessons

- Bilateral metachronous presentation of primary and secondary angiosarcoma, although extremely rare, should be considered in patients with a history of breast AS who develop new contralateral or ipsilateral lesions.
- Aggressive surveillance with cross-sectional imaging (CT/MRI) is warranted, as clinical signs can be subtle and disease progression rapid.
- Current adjuvant chemotherapy and radiotherapy appear insufficient to prevent distant metastases in high-grade angiosarcoma; novel therapeutic approaches (e.g., anti-angiogenic agents or immunotherapies) need to be explored.
- Obtaining written informed consent for publication is critical, especially for cases with extensive clinical documentation and images.

## 5 Communication and ethical considerations

Throughout the patient’s clinical course, the multidisciplinary team engaged in honest, stepwise, and compassionate communication with the patient and her family. At each critical juncture — the initial diagnosis of right breast angiosarcoma, the detection of a new contralateral lesion, and the eventual discovery of widespread metastases — the clinical team provided clear and balanced information regarding the nature of the disease, the rationale behind each treatment recommendation, and the expected prognosis. Treatment decisions were made through shared decision-making, with the patient’s performance status, personal values, and quality-of-life priorities being carefully considered. The patient expressed a strong desire to pursue all feasible therapeutic options, and her autonomy was fully respected. Psychological support services were offered throughout her treatment trajectory. This case underscores the importance of integrating ethical communication and patient-centered care into the management of rare and aggressive malignancies.

## 6 Patient perspective

(Adapted from discussions with the patient’s family and legal representative)

“When the lump first appeared in her right breast, she thought it was just a benign change. The diagnosis of angiosarcoma came as a complete shock. She was brave through the mastectomy and the chemotherapy, even though the side effects were very hard. When a new lump appeared in the left breast only a few months later, she felt desperate but still wanted to try every possible treatment. Her greatest wish was to see her children grow up. Even after the cancer spread, she never gave up hope. Her family wishes to share her story so that other patients might benefit from a better understanding of this devastating disease.”

## 7 Conclusion

Breast angiosarcoma (AS) is a rare malignancy with an aggressive clinical course, non-specific imaging findings, and the absence of standardized treatment guidelines. We report an exceptionally rare case of metachronous bilateral primary and secondary AS in a single patient. While bilateral primary angiosarcoma of the breast has been rarely reported (20), the present case is even more exceptional due to the subsequent development of secondary angiosarcoma in the previously affected breast. This unique presentation underscores the critical need for the identification of more specific diagnostic biomarkers and therapeutic targets to facilitate early detection and improve the dismal prognosis associated with this disease.
