# Supplementary material for: Case Report: Metachronous bilateral primary and secondary angiosarcoma of the breast
Source: Front Oncol. 2026 Sep 17;16:1883637. doi: 10.3389/fonc.2026.1883637 (PMC13626914; doi:10.3389/fonc.2026.1883637)
Supplement: Supplementary file 1 [file Supplementaryfile1.pdf]

CARE case report guidelines

CARE Checklist of information to include when writing a case report

(cc) BY-NC-ND

Topic	Item	Checklist item description	Reported on Line
Title	1	The diagnosis or intervention of primary focus followed by the words "case report"	Page 1, Title
Key Words	2	2 to 5 key words that identify diagnoses or interventions in this case report, including "case report"	Page 1, Keywords
Abstract (no references)	3a	Introduction: What is unique about this case and what does it add to the scientific literature?	Page 1, Introduction, last paragraph
	3b	Main symptoms and/or important clinical findings	Page 2, Initial presentation and diagnosis
	3c	The main diagnoses, therapeutic interventions, and outcomes	Pages 2–3, entire Case Presentation
	3d	Conclusion—What is the main "take-away" lesson(s) from this case?	Page 5, Take-away lessons; Page 6, Conclusion
Introduction	4	One or two paragraphs summarizing why this case is unique (**may include references**)	Page 1, Abstract
Patient Information	5a	De-identified patient specific information.	Page 2, Initial presentation (age, sex only)
	5b	Primary concerns and symptoms of the patient.	Page 2, Initial presentation
	5c	Medical, family, and psycho-social history including relevant genetic information	N/A
	5d	Relevant past interventions with outcomes	N/A
Clinical Findings	6	Describe significant physical examination (PE) and important clinical findings.	Page 2 (right breast); Page 3 (chest wall and axilla)
Timeline	7	Historical and current information from this episode of care organized as a timeline	Page 2, Timeline of key events; Figure 1 (Page 9)
Diagnostic Assessment	8a	Diagnostic testing (such as PE, laboratory testing, imaging, surveys).	Pages 2–3 (ultrasound, mammography, CT, MRI, biopsy, IHC)
	8b	Diagnostic challenges (such as access to testing, financial, or cultural)	N/A
	8c	Diagnosis (including other diagnoses considered)	Pages 2–3 (pathology and IHC); Page 5 (differentials)
	8d	Prognosis (such as staging in oncology) where applicable	Page 3 (Disease progression and outcome); Pages 5–6 (Discussion on prognosis)
Therapeutic Intervention	9a	Types of therapeutic intervention (such as pharmacologic, surgical, preve	Pages 2–3 (mastectomy, local excision, chemotherapy, radiotherapy, palliative surgery)
	9b	Administration of therapeutic intervention (such as dosage, strength, duration)	Page 2 (chemotherapy: 4 cycles ifosfamide+epirubicin; radiotherapy: 50 Gy/25 fractions)
	9c	Changes in therapeutic intervention (with rationale)	N/A
Follow-up and Outcomes	10a	Clinician and patient-assessed outcomes (if available)	Page 3, CT progression and patient death
	10b	Important follow-up diagnostic and other test results	Page 3, Disease progression and outcome (CT findings of metastases; see also Figure 7, Page 9)
	10c	Intervention adherence and tolerability (How was this asse	Page 2, grade IV bone marrow suppression
	10d	Adverse and unanticipated events	Page 2 (bone marrow suppression); Page 3 (rapid progression and death)
Discussion	11a	A scientific discussion of the strengths AND limitations associated with this case report	Page 4, Strengths and limitations
	11b	Discussion of the relevant medical literature **with references**.	Pages 4–5 (Discussion with literature); Pages 6–7 (References 1–20)
	11c	The scientific rationale for any conclusions (including assessment of possible cause	Pages 3–4, hypotheses on latency and genetic susceptibility
	11d	The primary "take-away" lessons of this case report (without references) in a one paragraph conclusion	Page 5, bullet points; Page 6, Conclusion paragraph
Patient Perspective	12	The patient should share their perspective in one to two paragraphs on the treatment(s) they received	Page 6, Patient perspective
Informed Consent	13	Did the patient give informed consent? Please provide if requested	Yes ☑ No ☐<br>Yes – Page 8, Informed Consent and Ethics statement
